# Sport Administrators' Perspectives on Advancing Safe Sport

**DOI:** 10.3389/fspor.2021.630071

**Published:** 2021-06-08

**Authors:** Joseph John Gurgis, Gretchen A. Kerr

**Affiliations:** Safe Sport Lab, Faculty of Kinesiology and Physical Education, University of Toronto, Toronto, ON, Canada

**Keywords:** safe sport, safeguarding sport, sport administrators, education, policy, research, monitoring, complaint mechanisms

## Abstract

Numerous international high-profile cases of athlete abuses have led to efforts to advance what has been termed “Safe Sport.” Sport and coaching organisations are urgently designing and implementing policies, procedures and programmes to advance a culture of safe sport. However, we posit that these endeavours are occurring without a conceptual framework about what constitutes safe sport or how to achieve it. Without a consistent conceptual framework for safe sport, prevention and intervention initiatives may not be fully realised. As such, the purpose of the study was to explore sport administrators' perspectives of how to advance safe sport. Given the leadership positions sport administrators hold, understanding their perspectives may be helpful in informing a framework to guide the development and implementation of safe sport strategies. Using a constructivist grounded theory approach, semi-structured interviews were conducted with 13 sport administrators from different sport and coaching organisations to elicit views on how best to advance safe sport. The findings indicated that a multi-faceted approach embracing multiple advancement strategies was reportedly essential for progressing safe sport. Specifically, the sport administrators recommended that sport organisations establish a universal framework of safe sport, design and implement education, implement and enforce policies, establish independent monitoring and complaint mechanisms and conduct research to ensure that advancement strategies are current and applicable. The participants suggested that these advancement strategies are necessary to evolve sport from a culture that embraces hegemonic masculine narratives, interpersonal violence and controlling coach–athlete relationships, to a culture of sport that extends the safe sport focus beyond the prevention of harm to the promotion of positive values and human rights. The findings were interpreted through a safeguarding lens to propose a framework for achieving safeguarding sport, defined by the prevention of harm and the promotion of positive values in sport.

## Introduction

International high-profile cases of athlete abuses have led to efforts to advance what has been termed “Safe Sport.” A substantial number of abuse scandals revealed internationally in sport, including, the Nassar case in the U.S. and the Barry Bennell case in the U.K., to name a few, have pressured sport leaders to develop and enforce safeguards (Nite and Nauright, 2020). As such, national and international sport and coaching organisations have developed initiatives, such as educational programmes and policies to advance a culture of safe sport—one free from abuse and harassment. For example, Safe Sport International (2019), an international collaborative agency committed to the global eradication of all types of abuse, harassment and violence committed against athletes of any age, provides current research, consulting services, and educational webinars to enhance safeguarding measures amongst sport stakeholders and organisations. The Coaching Association of Canada (2020), a national coaching body offering educational training and resources to support coach development in Canada, offers a variety of training programmes and safety-based policies focused on ethics, concussion awareness and maltreatment. Several other organisations exist, including the U.S. Center for SafeSport (2020), the Child Protection in Sport Unit in the United Kingdom (2020), Play by the Rules (2020) in Australia, and The International Olympic Committee (2020), all of which implement their own approaches in attempts to advance safe sport. These organisations vary in their roles and responsibilities with respect to safe sport, from providing information and serving as an advocacy body only (e.g., Play by the Rules) to addressing complaints of sexual abuse (e.g., U.S. SafeSport).

Despite the increased attention on safe sport initiatives in numerous countries around the world, the advancement of safe sport has been challenging. First, there is an absence of a generally accepted definition of safe sport or of a framework for understanding and advancing safe sport. For example, International Olympic Committee (2017) toolkit defined safe sport as safeguarding from harassment and abuse, Safe Sport International (2019) refers to protecting the welfare, safety and rights of all athletes and the U.S. Center for SafeSport (2020) refers to building a sport community where participants can work and learn together free of emotional, physical, and sexual abuse and misconduct. Canada's Sport for Life defines safe sport as the provision of a “training and competitive environment for athletes, coaches, officials, and volunteers that is free of abuse, harassment, and discrimination… Additionally, safety includes the physical aspect of the equipment and training practices” (Higgs et al., 2019, p. 11). These descriptions of safe sport highlight the variations in populations of interest–from athletes to all sport participants–and in focus—from protection from harm to protection of rights.

Without a consistent framework for safe sport, it follows that policies, programmes and practises to advance safe sport will also vary. Kerr and Kerr (2020) offered a critique of the interventions that have been implemented internationally to address safe sport and protect athletes from harm. As athlete maltreatment is a systemic issue requiring safeguarding interventions that extend from the individual to the organisational and societal levels (Kerr et al., 2019), Bronfenbrenner's (1979) Bioecological Systems Theory was used to address safeguarding strategies at each level of the theory. At the individual level, athletes' knowledge and awareness of safe sport-related topics are encouraged through the delivery of educational programmes. At the microsystem level, codes of conduct and educational programmes for stakeholders, such as coaches and parents, have been developed to enhance the interpersonal relationships between athletes and other stakeholders and to improve the conditions of the environment. The mesosystem, which focuses on the roles of organisations and institutions in positively influencing the conduct of sport stakeholders, has been addressed through the implementation of harassment and protection policies that preserve the physical and psychological welfares of athletes. At the exosystem level, emphasis is placed on the development of organisations responsible for and committed to advancing safe sport, such as U.S. Safe Sport. Finally, the macrosystem level, which considers the national, international and local policies, laws, regulations and sociocultural beliefs that are established to globally achieve safe sport, has been pursued through advocacy efforts, such as the International Olympic Committee's Consensus Statement on Abuse and Harassment (Mountjoy et al., 2016) and the accompanying toolkit (International Olympic Committee, 2017). The authors also highlighted the current weaknesses of the sport system with respect to its (in)ability to advance safe sport strategies at all levels of the model, including, for example, a lack of conceptual clarity including inconsistent definitions and descriptions of unsafe behaviour, educational programmes for athletes that are perceived as victim-blaming, policies that focus primarily on sexual abuse and neglect more commonly experienced forms of maltreatment, educational programmes that are not empirically or theoretically driven, ineffective monitoring and evaluation of programmes and difficulty disseminating programmes and information to a community of volunteers. The authors proposed that the “autonomous, self-regulating nature” of sport explains why sport is lagging in the area of child protection compared to other child-populated domains and why athletes remain silent about their harmful experiences (Kerr and Kerr, 2020, p. 98).

Several recommendations have been suggested to advance safe sport. For instance, Noble and Vermillion (2014) proposed to prevent maltreatment in youth sport programmes, “administrators and leaders must develop and implement stringent policies and procedures placing the safety of their youth participants as their main priority, and creating a culture of zero tolerance for any form of abusive behaviour” (p. 52). Mountjoy et al. (2016) suggested that to eliminate abuse from sport, “a systematic multiagency approach” that considers the design, implementation and evaluation of culturally-relevant, safe sport policies and procedures, education, and law enforcement strategies is required (p. 1019). Furthermore, Mountjoy et al. (2015) recommended “clearly defining inappropriate and violent behaviours in sport,” which may assist organisations with the adoption of proper safeguards in sport (p. 885). Having a unified understanding pertaining to which safeguards should be implemented by sport organisations may also ensure that there are consistent efforts to respond to unsafe practises.

The International Safeguarding Children in Sport Founders Group, comprised over 50 organisations, developed the International Safeguards for Children in Sport, including eight safeguards to protect children participating in sport from harm: (1) developing policy; (2) designing procedures for responding to safeguarding concerns; (3) provision of advice and support; (4) minimising risks to children; (5) identifying guidelines for behaviour; (6) recruiting, training and communicating; (7) working with partners and (8) monitoring and evaluating (Mountjoy et al., 2015). The safeguards are meant to “reflect international declarations, the United Nations Convention on the Rights of the Child, relevant legislation, government guidance, existing child protection/safeguarding standards and good practice” (Rhind and Owusu-Sekyere, 2018, p. 42). Furthermore, research that involved ongoing interviews and group discussions with organisation leads, as well as continuous feedback from the Founders Working Group, led to the creation of the “CHILDREN” framework, an acronym that stands for cultural sensitivity, holistic, incentives, leadership, dynamic, resources, engaging stakeholders, and networks, which should be considered when implementing the international safeguards (Mountjoy et al., 2015).

At the 2019 National Safe Sport Summit, hosted by the Coaching Association of Canada, current and retired national level athletes recommended a variety of strategies for advancing safe sport. Recommendations included: address all forms of maltreatment (rather than exclusively focusing on sexual misconduct); design and implement mandatory education for all sport stakeholders; prohibit all sexual relations and forced acts of intimacy between athletes and individuals in positions of power, such as coaches and support staff; increase the focus on athletes' holistic well-being; strengthen accountability measures; provide support and resources to victims of maltreatment and implement an independent regulatory body to investigate, respond to and adjudicate complaints and apply sanctions (Kerr et al., 2020, p. 76). At the time of writing this paper, Canada awaits a decision on a national independent mechanism to address concerns of athlete maltreatment, and only then will we know whether athletes' recommendations have been heeded.

Previous researchers have recommended that ensuring safe sport is the responsibility of all adults in the sport (Brackenridge, 2001; Kerr et al., 2019), and it may be argued that sport administrators hold a particularly important position of influence. Sport administrators have positions of power and authority over the operations of the organisation, including funding allocations, staffing decisions, implementation of policy and procedures, risk management and legal issues and accountability. Moreover, sport administrators have significant influence on the culture of the organisation by infusing values and priorities through communications, decision-making and implementation of policies; the sport administrator can determine whether the organisational climate is one that prioritises safe sport or performance excellence or revenue generation, as some examples. Given the responsibilities of sport administrators to set the tone of their organisation including which priorities are established and operationalised, monitored and evaluated, it is important to understand sport administrators' views on what is needed to advance safe sport. As such, the following study sought to understand sport administrators' perspectives of how to advance safe sport. Understanding sport administrators' perspectives may be helpful in informing a framework to guide the development and implementation of safe sport strategies.

## Materials and Methods

### Paradigmatic Position

This study adopts a constructivist paradigmatic position to further understand the perspectives of sport administrators regarding the advancement of safe sport. Constructivism upholds an interpretative worldview that advances the notion that reality is actively created by individual interactions with society and the environment (Rapmund, 1999; Kukla, 2000; Campbell, 2002), and in this way, interpretations of reality are culturally and socially influenced (Rapmund, 1999). A constructivist approach to conducting research with stakeholders, such as sport administrators, can illuminate the practicality of theoretical knowledge facilitating learning through the sharing of knowledge and experiences (Mesquita et al., 2014). The conceptualisation of strategies to advance safe sport was a codependent process facilitated by the negotiation of different topics between the participants and researchers.

#### Ontology

Research positioned within a constructivist paradigm embraces a relativist ontology (Guba and Lincoln, 1994; Lincoln et al., 2011). A relativist ontology posits that realities may be understood as several, imperceptible “mental constructions” that are constructed “experientially” and co-constructed “socially” (Guba and Lincoln, 1994, p. 110). A relativist ontology suggests that sport administrators' awareness of strategies to achieve safe sport is formulated through negotiated interactions with other stakeholders, the researchers and the environment in which they are immersed.

#### Epistemology

A constructivist paradigm assumes a transactional, subjectivist epistemology. Guba and Lincoln (1994) reported that the researcher and researched “are assumed to be interactively linked so that the “findings” are *literally created* as the investigation proceeds” (p. 111). Moreover, subjectivism acknowledges that separation “between the knower and the known” cannot exist “because all knowledge is constructed through a meaning making process in the mind of the knower” (Daly, 2007, p. 23). The epistemological assumptions of this study consider that sport administrators' knowledge of safe sport strategies is created through the recollection of prior safe-related experiences, personal experiences and through social interactions with other stakeholders and the researcher, which may influence the participants' recommendations of safe sport practises.

### Methodology

The following study utilised a grounded theory methodology to investigate the strategies that sport administrators recommend to advance safe sport. Grounded theory is defined as an inductive, methodical, and comparative style of conducting research for the purpose of theory development (Bryant and Charmaz, 2007). Researchers engage in an iterative process of transitioning between empirical data and emerging analysis; this process ensures that data analysis becomes increasingly more focused and theoretical (Bryant and Charmaz, 2007). Grounded theory research has been characterised with the following criteria: concurrent data collection and analysis, inductively developing analytic codes/categories, reliance on the constant comparison method, evolving theory throughout each stage of data collection/analysis, memo-writing, and theoretical sampling (Charmaz, 2006; Kenny and Fourie, 2015).

Methodologically, we adopted a constructivist grounded theory method (CGTM), which is an interpretative and iterative strand of grounded theory methodology that acknowledges the co-construction of knowledge between researcher and participant and perceives data analysis as the construction of multiple realities created through the negotiation of topics with the participants (Belgrave and Seide, 2019, p. 301). Charmaz, known for advancing the CGTM, suggested that this approach prioritises the phenomena of interest and interprets the data and analysis as being co-created through shared relationships and experiences between participants and other sources of data gathered from their surrounding environment (Charmaz, 2006). A CGTM acknowledges the interpretive nature of developing theory; this process encourages the researcher to be reflexive, flexible and creative in theorising data to interpret participants' experiences and knowledge (Charmaz, 2006).

### Participants

Thirteen sport administrators who held leadership positions within national and international sport and coaching organisations were recruited to share their views on how best to achieve safe sport. Sport administrators are at the forefront of designing and enforcing strategies that promote safe sport. Daube and Thomas (2016) acknowledged the social responsibility of sport administrators to monitor organisational behaviour and promote sport codes that protect athletes and promote healthy behaviours. Furthermore, given the limited understanding of the structural and social mechanisms in sport organisations through which athlete maltreatment is enabled and normalised (Roberts et al., 2020), it seemed critical to speak with sport administrators to understand their views on how to best respond to and mitigate the risks associated with unsafe and violent practises in sport.

To preserve confidentiality, a pseudonym has been assigned to each participant. Sport administrators are referred to as “SA”; to further distinguish between participants, they have also been assigned a numerical value, for example, SA1 and SA2, etc.

### Data Collection

The dialectical and interpretative nature of CGTM welcomes methods that elicit open dialogue. The following study included the use of in-depth semi-structured interviews. Although an interview guide was prepared in advance, we allowed space for unanticipated directions to the questions and responses. Charmaz (2006) advocated for using in-depth interviews to intimately explore the meanings participants attach to their shared experiences. To understand the ways in which we can advance safe sport, sport administrators were asked questions such as: “What strategies would you implement to achieve safe sport?” “What are the barriers to advancing safe sport?” and “What facilitators are required to advance safe sport?” Several probes were also used, such as “Please tell me more about that,” “Can you provide me with an example?” and “What were the positive and negative implications of that safe sport initiative?” The interviews ranged between 45 and 120 min; all interviews were audio-recorded with the participants' consent and transcribed verbatim.

Multiple sampling procedures are considered when recruiting participants for grounded theory research. The initial stages of the study relied on convenience sampling; participants who were accessible to the researcher and satisfied the participant criteria were asked to participate in the study. Morse (2007) suggested that the early phases of sampling aid in defining the scope, boundaries and trajectory of the study and research process. After preliminary data analysis, purposeful sampling was utilised to find participants who fell along the trajectory of the study (Jones et al., 2014). In the later stages of research, theoretical sampling was employed; this approach sought to collect relevant data through participants who were believed to contribute to the elaboration, development and refinement of emerging theory (Charmaz, 2006). For this project, participants were limited to those who met the age of consent and were affiliated with sport as an administrator.

### Data Analysis

Constant comparisons and memo-writing are often relied upon when analysing data in grounded theory research. Charmaz (2006) defined the constant comparative method as:

A method of analysis that generates successively more abstract concepts and theories through inductive processes of comparing data with data, data with category, category with category, and category with concept. Comparisons then constitute each stage of analytic development (p. 187).

At each stage of analysis, constant comparisons were made as part of the coding process. A CGTM includes two main phases of coding–initial and focused–followed by a process of theoretical coding (Charmaz, 2006). During initial coding, we thoroughly read through the transcript data line-by-line (Jones et al., 2014) and answered the questions, “What are these data a study of?” and “What do the data suggest?” (Charmaz, 2006, p. 47); answering these questions assisted in the naming of initial codes. Next, we engaged in a process of focused coding, which permitted us to develop more selective, directed and abstract codes (Charmaz, 2006). In this stage of analysis, focused codes develop into theoretically rich and integrative categories (Jones et al., 2014). The final theoretical codes depict potential relationships between categories of codes developed through focused coding (Charmaz, 2006). The grounded theory that emerges from the data is founded on the formation of the integrative theoretical codes (Charmaz, 2006; Jones et al., 2014).

Lastly, analysis occurred through a process of memo-writing, “… *the* fundamental process of researcher/data engagement that results in a “grounded” theory” (Lempert, 2007, p. 245). Memos were written with an analytical, rather than a descriptive, mindset (Jones et al., 2014). As the analysis progressed, literary resources were examined so that the theoretical underpinnings of other research could aid in identifying patterns within our dataset (Lempert, 2007).

### Ethical Considerations

The study protocol was reviewed and approved by the Research Ethics Board at the University of Toronto (approval number 37715). A detailed letter of information and consent form was provided to each sport administrator and reviewed prior to conducting the interview; the purpose of the letter of information was to highlight the participants' rights and the risks of participating in the study. Participants were required to provide written informed consent before the interview began. Participant confidentiality was assured given the social risks associated with portraying participants negatively or the disclosure of information that may jeopardise their public image or position or the reputation of the organisation they lead. To preserve confidentiality, all participants were assigned a pseudonym, and any personally identifiable information was omitted from the study.

## Results

### Recommendations for Advancing Safe Sport

Recommendations made by the sport administrators to advance safe sport included: constructing a universal framework of safe sport for all sport organisations to adopt; the development of safe sport education, policy implementation and enforcement; the establishment of independent monitoring and complaint mechanisms and ongoing research to support the development and refinement of safe sport policies and procedures. The sport administrators perceived these strategies as being essential in shifting the culture of sport towards one that is committed to the prevention of harm and values-based.

The subsequent sections will elaborate on the sport administrators' recommendations for advancing safe sport.

#### Advancing Safe Sport Through an Established Framework of Safe Sport

Participants claimed that the absence of a universal understanding and definition of safe sport weakens efforts to advance it. As such, participants recommended that a generally agreed upon definition or framework of what safe sport is be developed, as highlighted by SA2, “I think that there is a need for a [safe sport] framework that would apply to all sport, so we understand what exactly we're all talking about.” SA9 acknowledged the importance of having one, consistent definition because the varying interpretations of safety in sport have made it difficult to advance a culture of safety:

Safe sport was originally focusing on preventing abuse, but now it has expanded to include so many other factors. Is the exclusion of trans-athletes a safe sport topic? Does the inaccessibility at arenas represent a safe sport issue? I would say yes, but that's not the common stance held by my peers… I agree, I do think we need a unified definition. It provides us [sport administrators] direction with what is needed to improve safety in sport.

SA9 recognised the significant developments of safe sport over time, and consequently, it has become increasingly difficult for sport administrators to advance safe sport because the topics included are ever-changing. SA11 also expressed the need for a consistent definition of unacceptable conduct across all sports:

The idea of sport, very generally, is flawed. Within the greater scheme of sport, you have multiple sports that have their own system of right and wrong. So, [ice] hockey tells you it's okay to drop the gloves to settle an issue, but volleyball says otherwise. So, what does my kid learn when he's in a conflict? It's okay to use your fists or your words to resolve it. That's non-sense. You can't have millions of policies and programmes that define safety differently from sport to sport because they'll all at some point end up contradicting each other and we won't ever actually achieve safety. Every sport needs to be consistent. Violence is wrong, abuse is wrong, these are the repercussions. When individual sports start to align their stance on safety, sport as a whole will start to eliminate these broader issues of abuse and so on. (SA11)

Similarly, SA10 stated:

I don't think there's one definition of safe sport, and that's the problem… It's a continuous type of work that identifies different issues that can hurt an athlete, hinder development, or enjoyment of sport. With so many issues in sport we've developed so many definitions, so how do we help anyone if our understanding is continuously broadening?

The participants referred to the changing field of safe sport as presenting continuous challenges; as sport administrators commit to making sport safer, they need to explore the issues that contribute to an unsafe environment in an ongoing way. SA12 agreed that a unified philosophy of safe sport is required and, like other participants, suggested that safe sport should include the reduction of physical and psychological harm or unsafe practises and also extend to include the benefits of sport participation:

I think there's a tendency to go to the headlines and to the problematic behaviours and for sure there is a need to prevent those behaviours so that has got to be a major area of focus, but I think it really limits the impact of the whole safe sport concept if it doesn't describe those benefits and the positive things that come out of sport. I think part of what's missing in a lot of cases is the lack of a clear philosophy of sport or a philosophy of athlete-centred sport… we need that universal philosophy of safe sport which isn't all about the negatives. It's about the positives, the benefits, and the reasons why we do this and the importance of being athlete-centred. I think that's something that's missing.

SA12 acknowledged the importance of identifying the problematic behaviours prevalent in sport; however, SA12 recommended advancing a universal philosophy of safe sport that extends beyond harm-based definitions and focuses on the benefits of sport and the promotion of athlete-centred values.

#### Advancing Safe Sport Through Education

All participants agreed that offering education around safe sport was integral to creating a physically and psychologically safe environment for all participants. According to SA10, “The answer is education. I think we need to spend much, much more time educating… I think everybody needs to be educated and everybody needs to comply with safe sport requirements.” Whereas, most safe sport education targets coaches, SA10 suggested that additional education be developed and made compulsory for all participants in sport. For other participants, education was often recommended solely for coaches:

I think part of the challenge with the coaches is to educate them on the harm that they are doing to the individual on the emotional, psychological, and physical level that is detrimental long-term. To make them understand that whatever they learned as young athletes themselves or whatever they witnessed and thought that's how you make people suffer or whatever, that it is causing very serious harm and it is not as effective in getting the result they are after because to be more positive, to be more supportive, to allow for more enjoyment of the sport, all those things will actually elicit much greater performances from the athletes… The education part is really important. Letting them know that these kinds of activities constitute physical or psychological harm… Education about these things is really important first and foremost because some of these definitions of abuse are going to challenge people's view of what they felt was acceptable and normal coaching techniques. (SA2)

SA12 further supported the notion that coaches need education to encourage self-examination and reflection:

I think education programmes are important for coaches to better analyse their own behaviours and realise what some of the problems are and what some of the things are that they can do to manage the problems… programmes that encourage self-reflection allow some of these coaches to realise how problematic their behaviours are.

SA12 elaborated on the importance of safe sport education to move beyond awareness-raising to address ways of facilitating behaviour change:

I do think the education programmes don't go far enough. I think they convey information but they're not going to the point of really achieving behavioural change. There is far more work that has to be done in terms of learning and supporting coaches in the environment to really bring about behavioural change.

Numerous educational topics were recommended by sport administrators to be included in safe sport education, including “policy, prevention, about different types of misuse of power, education about how to report and when to report” (SA10). SA1 suggested topics related to “all the “-isms” should be addressed [in safe sport education]—racism, ableism, sexism, classism, ageism. Anytime someone is discriminated against, that produces an unsafe experience. People need to know that.” SA5 acknowledged the growing concern of mental health issues and the importance of educating coaches on how to recognise these issues:

Coaching today has to change given the mental health issues. For example, say they have a student-athlete with significant mental health issues on their team, coaches typically don't know how to deal with that type of athlete… the coaches often don't know what's going on with the athlete. The athlete may be in a better situation to take time off the sport to get mentally better, but they don't want to, and they end up staying with the team. The coaches don't know how to deal with that situation, they assume the player isn't strong enough to play and end up cutting them. Well, that creates more mental health issues. It can be dangerous. It is a really complex issue. The coaches are struggling with how to deal with that and need to be informed. It impacts the team dynamic, performance, and can be mentally unsafe for athletes.

Finally, a few participants recommended that safe sport education addresses the positive side of sport. SA13 explained, “we focus a lot on the terrible things happening in sport and not enough on the good that can come from sport. If we educate others on the positives, then maybe that becomes the new norm.” Similarly, SA9 suggested that safe sport education that is values-based would enable the safe sport movement to thrive:

These conversations about safe sport, they're very reactive. We've had terrible acts of abuse that have tainted the image of sport and now everyone is frantically creating education, policies, presentations, you name it, to increase people's awareness about the dangers of sport. Now, whenever you think safe sport, you think of child sexual abuse or harassment. We should be focusing on reminding people about the good in sport because let's face it, you would be a fool to continue to participate in sport after hearing about safe sport. Safe sport is abuse? Safe sport is harmful? Safe sport is dangerous? Yeah, no thank you. Instead, safe sport is healthy? Safe sport is fun? Safe sport is fair? Yup, I'll take that. Safe sport would thrive if we thought that way because people want to be part of the solution, not the problem. They want to identify with something positive, not negative.

Both SA13 and SA9 recognised the limitations of safe sport education that focuses solely on the prevention and reduction of harm and the value of positioning safe sport education to promote the positive values of healthy, fair and safe sport experiences.

#### Advancing Safe Sport Through Policy Implementation and Enforcement

The sport administrators acknowledged the importance of policies to foster a safe sport environment; however, the benefits of this advancement strategy were reportedly contingent on many factors. First, participants emphasised that policies need to clearly define unsafe conduct and consequences for breaching policies. For example, SA7 described the policies defining unacceptable behaviour and related consequences that coaches are expected to adhere to:

There is zero tolerance for abuse, sexual assault or harassment, some statements about drugs and alcohol, abuse of power and abuse of players or officials. There is a whole series of them. We've developed automatic sanction charts for all our league sports, so coaches and athletes know that there is no grey area. You do this, this is your suspension or suspension and fine or suspension and fine and review. They can actually lose their privilege of playing for us this season.

The clear delineation of consequences appears to give safe sport policies traction. Additional consequences were suggested in response to violating policies. For example, SA13 advised on cutting funding from sport programmes or organisations that fail to comply with policies: “consequences of not being compliant, probably withholding funding, seems to crack the whip in sport in Canada.” In the absence of consequences, participants believed that policies are rendered useless. SA13 continued:

You can have a nice policy document but if no one ever actually checks on whether you are abiding by it and there are no consequences as a result of a breaching that code of conduct, then it might as well not be there.

Participants identified weaknesses in the enforcement of existing policies as well:

I think enforcement of policies is quite weak because coaching isn't a profession and it isn't a regulated activity. Whatever policies are put in place tend to be dependent on goodwill not on a real enforcement mechanism so people can avoid a lot of the policies if they choose to. So, I think there is work that needs to be done on enforcement and regulation. (SA12)

SA1 reinforced the notion that for policies to be effective, they need to be linked with enforcement processes:

Policies are great. They don't do anything in and of themselves. It has to be policies with implementation plans… I don't think it's a strength in our sport system. I think policies are checkboxes for our funders. Some organisations are great at taking these policies and bringing them to life. I do think organisations need help also with the implementation.

The perception that policies are mere “checkboxes” for sport organisations suggests that in the absence of enforcement strategies, safe sport policies are ineffective.

#### Advancing Safe Sport Through Independent Monitoring and Complaint Mechanisms

Participants recommended advancing safe sport through the development of an unbiased, centralised, independent body, separate from the sport organisation, that would be responsible for conducting investigations on complaints of safe sport-related issues. SA13 stated:

I think a truly neutral system that involves reporting, like a triage investigating mechanism that could also refer third-party support services that are not just to handle that complaint. If there are mental health resources that are necessary, child protection etc… There is a bit of a web that happens depending on what that triage looks like but the investigation that ensues -that is fully independent and coming from a third-party and then ultimately a tribunal or adjudication type process that can actually look and potentially sanction individuals or parties for non-compliance.

SA13 continued:

I think it needs to be a fully independent neutral third-party mechanism that will uniformly enforce the code and make sure that organisations are compliant with it, that everybody has the same expectations and understandings and then oversee any kind of investigation that happens as a result of any kind of breach or report and then potentially have actual discipline proceedings or be able to enforce sanctions.

According to the participants, the development of an independent body not only ensures that reports are handled impartially but also allows for specific sanctions to be placed on non-compliant sport programmes and organisations. SA10 expressed:

I think its crucially important to have a centralised body that deals with [safe sport]… We want medals, we want trophies, we want prize money. I think the next step is making sure that this comes second after making sure that everybody is safe… having a centre focusing on safety would be best.

Similarly, SA1 recommended having external third parties to police stakeholders in sport and hold them accountable for their actions:

The idea of third parties and external parties, I think is really relevant. I personally advocate for the idea of having an independent body…The accessibility of other supports and other observers or whatever the right term is or an independent officer to be there. I think the trick being that they have a policing role sort of to speak… I think it holds people accountable.

SA7 agreed that the behaviours of coaches should be monitored as a way of advancing safe sport: “If you are worried about safe sport and how coaches communicate with athletes and deal with mental and physical and emotional abuse, why is there no observation level? Why are we not observing coaches in the training setting?”

Participants recognised how sport organisations may be ill-equipped to investigate reports of harm, and thus intervention from an independent third-party may relieve sport administrators of tasks they are unfit to execute. SA13 explained:

I think what the messaging should be for national sport organisations is that an independent body will take actually like a lot of issues off their hands too… I know national sport organisations like [Canadian sport organisation] say that they don't want to deal with those issues. They want an independent third-party to deal with these issues. They don't want the liability. They know they are not specialised or capable. They are very happy to say “please independent third-party step in and investigate and tell us what to do here.”

SA13 acknowledged how it would be legally wise for sport organisations to consider the aid of an independent third-party. Similarly, SA9 agreed that an independent body would be beneficial to support sport organisations who often seem unqualified to effectively investigate cases of abuse:

I think the increased media attention on sexual abuse has pressured organisations to step up when it comes to responding to cases of abuse. And yes, many organisations have not been fully transparent, but for many others it's a capacity issue. We don't have the support, resources, funding, time to investigate every report of abuse… I'm also not qualified to respond to these issues, and neither are many others on my team. We're volunteers. We are qualified in other ways… definitely, I think it would be advantageous for an independent body to be established in sport. They can focus on that aspect of sport and we can continue to focus on ours.

In addition to not being qualified, SA1 acknowledged that internal investigations of abuse are often unworkable due to limited sources of support; as such, it would be beneficial for sport to establish an independent system to investigate reports of abuse.

#### Advancing Safe Sport Through Research

Although not as prominent as the other recommended strategies, a few participants understood the importance of research informing the advancement of current safe sport strategies. SA4 stated:

Quality education needs to do a better job of being more responsive to research so we can do a better job of keeping materials, trainers and learning facilitators up to date. The whole reason we are doing this is so that participants of all ages are getting the best experience. We need to ensure that they have the best material being taught to them or being shown to them or learn from so that the athletes are getting the most quality experience. I would say the one thing is becoming more responsive to the changing of research and times.

Similarly, SA1 highlighted the importance of research:

The research and evidence in this area is extremely important to us. If I don't have evidence to back things up, I can't say it. I can talk anecdotally. I can say this is logical but having the ability to look at… research and say abuse and harassment is gendered. We know in society violence against women is manifesting in sport and we actually have evidence of that. What do we think of that then? We can have a conversation then. We can create policies that achieve real change because we understand the evidence driving those policies.

Finally, it was recognised that further research is required to improve the current reporting mechanisms in place within Canadian sport:

There's been countless failed attempts by organisations trying to respond to reports of abuse. An independent reporting system would make the most sense for athletes, but the logistics of it need to be explored further. I think the research will really justify to organisations why this is an important step in the field. It's not to expose the organisation of wrongdoing, but really aid sport organisations in fully embracing safe sport and protect athletes. (SA13)

#### Shifting Towards a Safe Sport Culture

The participants perceived their recommendations as necessary to stimulate a cultural shift in sport. Specifically, the establishment of a universal framework, development of safe sport education, implementation and enforcement of policies, the provision of independent complaint mechanisms and ongoing research are believed to be fundamental to challenging the current sport culture in which harmful behaviours are normalised. For example, SA10 highlighted the need for a cultural shift and cited the current culture as a barrier to achieving safe sport:

I think the main reason is cultural. You know what they call “old school”? I hate this word. I've heard it so many times. It's resistance to change because people think the way they did it is best… there's lots of good coaches… They got individuals to the Olympics, they got gold medals, but that doesn't mean that's the right way to do it. They come and tell you, “Well don't tell me how to behave. I have five individuals from my career that went to Summer Olympics. Who are you to tell me how to coach my athlete?” I think the resistance is from that old school type of education. It's realising that you can still achieve the same or better results, which we know by research already, without applying those techniques. And once you apply those [old] techniques, the athlete might win a medal, but you scar them for the rest of their life.

According to SA10, education is integral to advance a culture of sport that moves beyond the traditional style of controlling coaching. SA11 alluded to the controlling culture of sport as well and suggested that the control stems from the military and hypermasculine roots of sport:

I think that the culture, the background of sport coming from a military tradition and male-dominated area, it's been one of the areas that has been the slowest to change. It's really been going from a master to the athlete and then a lot of the athletes are getting into coaching and they're just repeating their own experience. The culture is lagging behind in a lot of changes… it's hard to educate and change…

A cultural shift was also identified as being important given the evidence of the oppressive culture, specifically towards women and individuals who identify with the LGBTQ community:

People are targeted with homophobic slurs as a way of trying to keep them in their place and minimise their power. This was as much reflected in sport and perhaps more because it's a hypermasculine space or has been traditionally more than other areas in society and so as research has shown… women in sport have been significantly impacted by harassment and discrimination and overall a culture of oppression where they don't feel like they belong… anecdotally we hear a lot from women about practises and precedence and attitude that would reflect a lot of hostility towards women's involvement or the women's involvement being tolerated to a certain point and certain women's involvement is seen less [as] acceptable than others… it does create an atmosphere for many women where they are made to feel that they are unwelcomed and certainly some say discriminated against in sport.

According to the participants, the current culture of sport must shift away from being oppressive, hypermasculine, violent, autocratic, and discriminatory.

The participants used several descriptors to articulate what they envisioned to be the outcomes of successfully advancing safe sport through the recommended strategies. Specifically, participants referred to values, such as ethical, open, respectful, welcoming, accepting, fair, fun, safe, inclusive, equal, empowering, holistic, humane and free, to describe what safe sport would look like if a cultural shift is successfully achieved. For example, SA2 suggested values that are foundational to real safe sport experiences:

A true safe sport experience would demonstrate sport principles of safety, respect, fairness, fun, inclusion… when those values are being promoted, highlighted, supported by the sport organisation, there is going to be less chance of us being involved in unsafe practises.

SA2 elaborated:

If sport organisations at the community level, up to the national level, are ensuring that the sport experience they provide is driven by sport principles of safety, respect, fairness, fun, inclusion, then there is going to be less chance of us being involved in unsafe practises and an increased chance of us transforming sport into an endeavour that prioritises the safety and needs of athletes. People in the sport sector need to be reminded of these positive values.

SA2 also recognised the potential for safe sport to build communities and strengthen the national sport identity:

Safe sport experiences could prevent the bad things from happening but can also promote the good. It will help instil character in our kids, it will strengthen our communities, sport organisations and neighbours will come together with their kids in sport, relationships will be built, social capital will be built, and many positive things will come from that… it will increase the base of participation in sport, it will increase the likelihood of creating greater excellence in sport and so on the world stage, we would have more athletes representing Canada. That's why a good sport experience or a safe sport experience is so important because it maximises those positive benefits.

Inclusion was referred to by participants as an aspect of safe sport. As an example, SA1 described safe sport policies better supporting transgendered athletes:

As we have come to understand gender identity and the sport model, you're either male or female based on your physical anatomy. We know that doesn't really explain the human condition, so we have helped develop policy in place for sport to use to make their sport more safe and welcoming for trans persons… it is about the safety of those individuals so that they can participate in sport in a safe and welcoming way and not be subject to any kind of discrimination or bullying or harassment based on their gender identity.

An ideal safe sport environment is an inclusive one—one in which inclusion expands beyond the acceptance of participants with distinct gender identities to include participants with varying abilities:

Access should be a fundamental right but because it's not built into a lot of our culture and behaviours, there are a lot of barriers. I think a lot of our Para-athletes end up normalising that some of the stuff they experience a lack of access to is okay. To make it, whether it is dealing with their disability and/or the lack of environment that is supportive, they need to work twice as hard… Do I think some of the information is readily available to our coaches? No… It just hasn't been part of the safe sport conversation, but it should be because without it, you end up losing out on some great athletes. (SA3)

SA3 continued, explaining that an ideal safe sport environment would extend beyond the prevention of harm to fostering a quality environment, defined as accessible, welcoming and inclusive:

Safe has a really basic, very minimalist view in my opinion for what we should be looking for… One of my concerns in a lot of this safe sport work is that it's very gap or issue oriented and for me, a safe, welcoming and inclusive system is way more than the absence of these issues. Right now, it feels like a lot of our strategies are focused on the reduction of harm or filling in the gaps as identifying with these areas and I am thinking okay, but it's like performance. It will neutralise it. It might stop it but it's kind of flat line…I think that's where some of the discussion needs to go so people understand the importance of these processes in creating an accessible and inclusive quality environment for all.

## Discussion

The purpose of this study was to explore sport administrators' perspectives on advancing safe sport. Analyses of the interview data indicate that sport administrators believe a number of strategies to advance a culture of safe sport are needed, including: establishing a universal framework, developing safe sport education for all, implementing and ensuring compliance with policies, creating independent monitoring and complaint mechanisms and researching safe sport to ensure that current programmes, policies and procedures are relevant. Additionally, the findings indicate that the effective implementation of these advancement strategies is perceived as fundamental to ensuring a needed cultural shift in sport, one that is characterised by the achievement of ideal, safe sport-related outcomes, such as inclusion, accessibility, fairness, safety, and human rights.

Interestingly, the strategies offered by the sport administrators already exist to varying degrees within the current landscape of safe sport. Sport organisations previously mentioned, such as the Coaching Association of Canada (2020), International Olympic Committee (2017), the U.S. Center for SafeSport (2020), and the U.K. Child Protection in Sport Unit (2020), have developed safe sport education addressing a range of topics, implemented safe sport policies focusing on the prevention of harm, established reporting measures in response to abuse and referenced safe sport-related research in their programmes and materials. Furthermore, the recommendations made by sport administrators are consistent with the findings by Noble and Vermillion (2014) that sport administrators recognise the importance of implementing policies on reporting maltreatment and ensuring employees receive adequate training that enhances their awareness of different types of maltreatment that manifest in sport. Wurtele (2012) further supports that participation in education is critical in preventing maltreatment and may influence positive changes in organisational culture insofar that sport administrators recommend and advance policies and procedures to enhance the safety of young and vulnerable participants. The findings of the current study also align with suggestions made by Mountjoy et al. (2015, 2016) to establish a framework to better protect athletes in sport and to advance policies and education. Specifically, the sport administrators in the current study acknowledged that clearly defining safe sport-related behaviours will aid sport organisations in understanding which safeguards are most appropriate to protect athletes from harm. The recommendations made by sport administrators are consistent with the existing advancement strategies and suggest that the participants are aware of the shortcomings associated with the current methods employed to achieve safe sport.

However, the study's findings differ from those of Mountjoy et al. (2015), who recommended defining the inappropriate and violent behaviours of sport to effectively advance safeguards. Based upon the reported positive effects of successfully advancing a safe sport culture, this study supports the notion that safe sport frameworks extend beyond definitions of harm prevention to include the optimisation of the sport experience through the promotion of positive values. Therefore, the recommended strategies would be designed and enforced not only to solely prevent harm but also to promote a culture of sport that is inclusive, accessible, welcoming and safe for all participants. The positive effects of advancing safe sport conveyed by the sport administrators in the current study are consistent with interpretations of values-based sport. Values-based sport represents an organisation's commitment towards establishing a sport system defined by the ideas of fairness, excellence, fun and inclusion (Public Policy Forum, 2019). Values-based sport allows all participants to experience the range of physical, emotional and social benefits sport has to offer and ensures that policies, programmes and procedures are designed to eradicate unethical issues corrupting the integrity of sport while simultaneously striving to improve the sport experience for all stakeholders of sport (Public Policy Forum, 2019).

The current participants' descriptions of an ideal safe sport environment are also congruent with the United Nations Convention on the Rights of the Child given their references to the recognition and promotion of human rights and the commitment to safeguarding children from all types of abuses and harm (Office of the United Nations High Commissioner for Human Rights, 2020). These findings are important because they extend the original and more commonly accepted purpose of safe sport of preventing maltreatment of athletes to the promotion of human rights and the recognition of sport's potential to contribute to optimal development of individuals, communities and societies. The promotion of human rights is a defining feature of the term safeguarding. The National Society for the Prevention of Cruelty to Children (NSPCC) in the United Kingdom describes safeguarding as: protecting children from abuse and maltreatment, preventing harm to children's health or development, ensuring children grow up with the provision of safe and effective care and taking action to enable all children and young people to have the best outcomes (National Society for the Prevention of Cruelty to Children, 2020). Safeguarding encompasses the benefits of safe sport (i.e., prevention of harm) and is driven by the promotion of positive values and human rights in sport. To achieve safe sport, therefore, sport organisations must develop education, policies, complaint mechanisms and research that not only prevent harm in sport but also advance values, such as inclusion, fairness, ethics and accessibility, as recommended by the participants. Interestingly, the term “safeguarding” has traditionally been used in the U.K. but is not the norm in other countries. Even in international sport organisations, such as the International Olympic Committee and Safe Sport International, the term safe sport is used rather than safeguarding.

The findings suggest that the sport administrators' recommendations are more congruent with advancing safeguarding than safe sport. The participants' reports are interpreted to indicate that the prevention of harm framework that largely characterises safe sport initiatives is limited and should expand to include the promotion of positive values to reflect the desired cultural shift towards safeguarding sport. Although some sport organisations acknowledge in their conceptualisation of safe sport the importance of promoting positive values, the safe sport strategies implemented by these organisations fail to exemplify these. It may be that sport organisations assume that strategies employed to prevent harm will also achieve the positive outcomes of inclusion, accessibility and adherence to human rights. As such, organisations may overlook the importance of designing and implementing advancement strategies to achieve these safeguarding outcomes distinctively and instead employ preventative strategies assuming the concurrent achievement of harm prevention and values-based sport. While values-based sport may be associated with the positive by-product of preventing harm, the reverse is not true. In other words, a focus on the prevention of harm exclusively will not guarantee the positive benefits that emerge from values-based sport, but a focus on values-based sport will inherently include the prevention of harm. This may explain why the sport administrators recommended strategies that currently exist within the safe sport landscape; these strategies are important but ineffective if grounded within principles of harm prevention rather than being values-driven.

**Figure 1:**
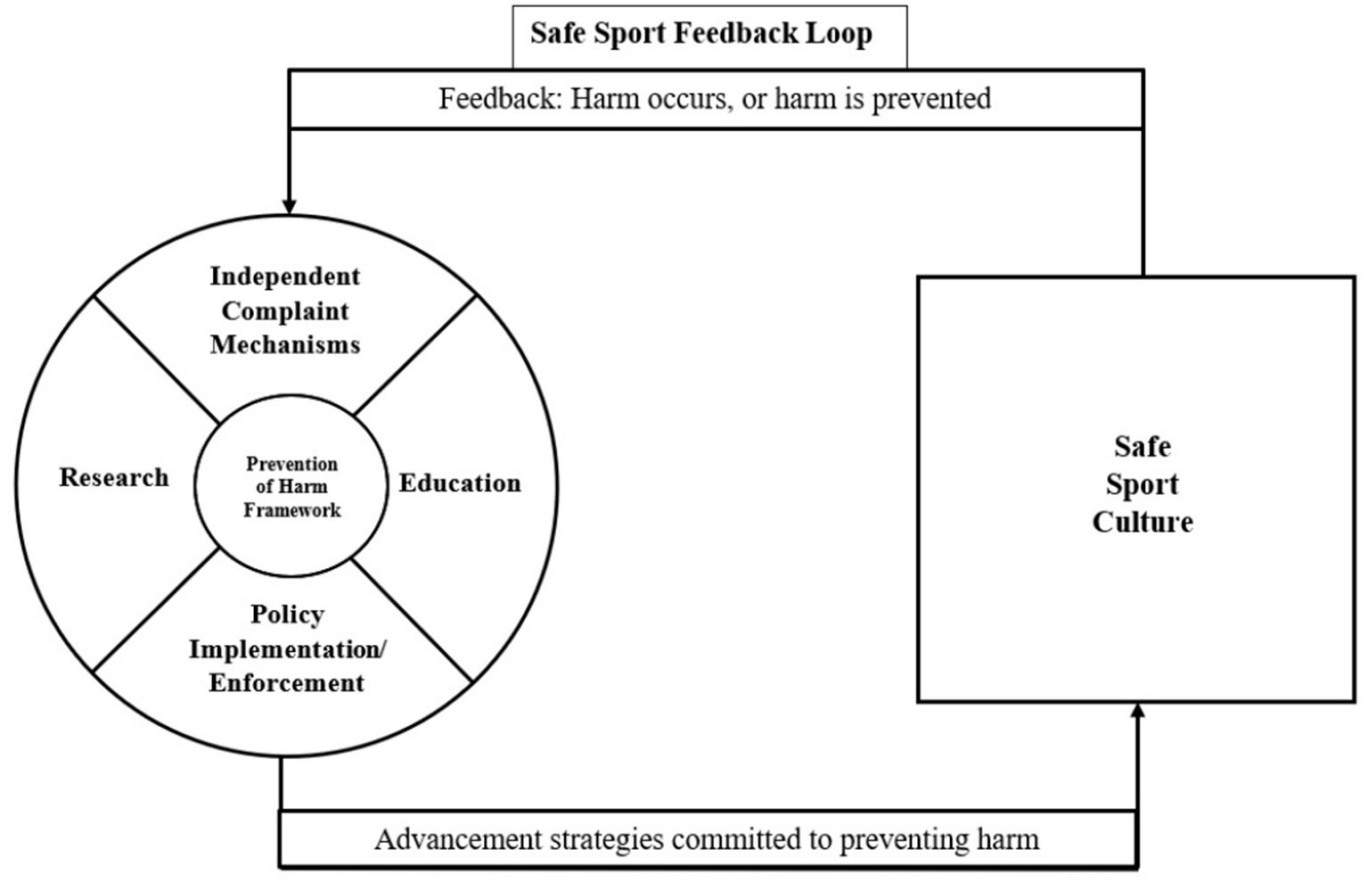
Prevention of harm framework for safe sport.

**Figure 2:**
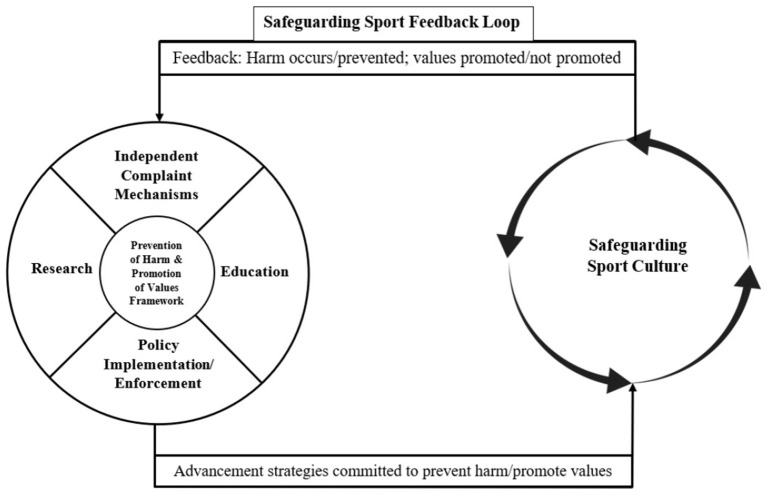
Values-based framework for safeguarding sport.

Informed by the data and research in the areas of safe sport and safeguarding, we propose a model that illustrates the current status of the safe sport landscape that reflects a prevention of harm approach (Figure 1). This is followed by an additional figure, which illustrates a values-based approach to safeguarding athletes (Figure 2).

### Prevention of Harm Framework for Safe Sport

The development and execution of the advancement strategies recommended by the sport administrators function collectively and interactively to reinforce a framework of prevention; all education, research, policies, independent monitoring and complaint mechanisms are designed to prevent harm in sport, thus contributing to a safe sport culture. The safe sport culture is depicted within a square to illustrate the confined interpretations of harm prevention that have largely defined safe sport. When a sport organisation employs these strategies, the success of these strategies is assumingly measured by reduced rates of harm within the sport organisation. For example, if there is a report of sexual abuse within a sport organisation, the strategies associated with safe sport may adjust to further prevent future acts of sexual abuse. The advancement strategies are continuously modified based upon the feedback received from the sport context (i.e., whether harm is occurring or not), and the feedback ensures that strategies are continuously refined to reinforce the prevention of harm framework. However, the findings of the current study suggest that a shift from a harm prevention towards a values-driven approach is needed. This is depicted in Figure 2.

### Values-Based Framework for Safeguarding Sport

The values-based safeguarding framework is a representation of the cultural shift advocated by the participants. Education, research, policies and independent complaint mechanisms are designed and implemented to achieve a safeguarding sport culture, characterised by the prevention of harm and the promotion of values, such as inclusion, safety, fairness, accessibility, and human rights. Similar to the prevention framework for safe sport, the advancement strategies in the values-based framework for safeguarding are interconnected to the extent that the development and modification of one strategy influences the others to some degree. However, in this framework, the advancement strategies reinforce both harm prevention and promotion of values-based sport. The safeguarding sport culture is depicted as an evolving circle to illustrate the everchanging, growing culture of safeguarding sport; this suggests that discourses of values-based sport related to concepts of inclusion, accessibility and human rights in sport will continuously evolve relative to societal changes and emerging research in these particular fields of interest.

Sport administrators' support of shifting towards a safeguarding sport culture demonstrates a commitment to confront and disassemble traditional and prevailing beliefs in sport, such as hegemonic masculine norms, win-at-all-costs and controlling coaching strategies (Hughes and Coakley, 1991; Silva et al., 2012; Stebbings et al., 2015). Preventing experiences of maltreatment will require a different set of prevailing assumptions, a notion supported by the current sport administrators' claims that a culture reflective of inclusion, accessibility and human rights is needed. By grounding advancement strategies in the principles of safeguarding, a cultural shift to the promotion of positive values and human rights in sport will be promoted.

This study was limited by exclusively investigating sport administrators' recommendations for advancing safe sport. Inquiring about the perspectives and recommendations from one stakeholder group may limit our understanding, and the recruitment of diverse participants (e.g., Black, Indigenous or LGBTQ) may elicit recommendations for advancing safe sport that have yet to be considered in the literature. Given the sensitivity of the topic area, the participants' perspectives may have been influenced by a social desirability bias, whereby participants' responses reflect the assumed interests of the researcher, rather than their actual personal views. Additionally, there was limited socio-demographic diversity in the sample, and only one method of inquiry was utilised to collect data. To advance safe sport, it is integral to explore the perspectives of other stakeholder groups, especially those of athletes, which are often missing in discourses of safe sport. Moreover, researchers should further explore ways to shift the culture of sport from a prevention of harm to a values-based approach in which human rights guide the design and implementation of sport. Finally, the recommended modes of intervention should be piloted and assessed within subcultures of sport to understand how these strategies, when grounded within a framework of safeguarding, affect the perspectives and behaviours of stakeholders and the welfare of participating athletes.

## Conclusions

The purpose of this study was to explore sport administrators' perspectives on strategies to advance safe sport. The findings indicated recommendations for a consistent framework for safe sport, education, research, policy implementation and enforcement and an independent complaint mechanism. These strategies are consistent with findings from previous studies and, interestingly, are already in existence across multiple sport organisations. We speculate that sport administrators have recommended strategies that are currently in place because the focus of these existing strategies is inadequate. More specifically, most sport organisations implement strategies that focus on the prevention of harm, but the sport administrators in the current study advocated for strategies that focus on the promotion of inclusion, equity, accessibility, and human rights, consistent with a values-based approach to sport. Such an approach is congruent with the notion of safeguarding that reflects both ensuring safety as well as the adherence to human rights.

## Data Availability Statement

The original contributions presented in the study are included in the article/supplementary material, further inquiries can be directed to the corresponding author.

## Ethics Statement

The studies involving human participants were reviewed and approved by University of Toronto Research Ethics Board. The patients/participants provided their written informed consent to participate in this study.

## Author Contributions

JG collected and analysed the data, wrote each section, and edited the manuscript. GK contributed to the analysis and writing and thoroughly edited the paper. JG and GK collaboratively conceptualised the study. Both authors contributed to the article and approved the submitted version.

## Conflict of Interest

The authors declare that the research was conducted in the absence of any commercial or financial relationships that could be construed as a potential conflict of interest.
